# Evaluation of the Effectiveness of a Novel Brain and Vestibular Rehabilitation Treatment Modality in PTSD Patients Who have Suffered Combat-Related Traumatic Brain Injuries

**DOI:** 10.3389/fpubh.2015.00015

**Published:** 2015-02-04

**Authors:** Frederick R. Carrick, Kate McLellan, J. Brandon Brock, Cagan Randall, Elena Oggero

**Affiliations:** ^1^Neurology, Carrick Brain Centers, Dallas, TX, USA; ^2^Neurology, Carrick Institute, Cape Canaveral, FL, USA; ^3^Harvard Medical School Global Clinical Scholars Research Training (GCSRT), Boston, MA, USA; ^4^Electrical and Computer Engineering Department, University of Wyoming, Laramie, WY, USA

**Keywords:** PTSD, vestibular rehabilitation, off vertical axis rotation, DSM-IV CAPS, brain

## Abstract

**Introduction:** Blast-related head injuries are among the most prevalent injuries suffered by military personnel deployed in combat and mild traumatic brain injury (mTBI) or concussion on the battlefield in Iraq/Afghanistan has resulted in its designation as a “signature injury.” Vestibular complaints are the most frequent sequelae of mTBI, and vestibular rehabilitation (VR) has been established as the most important treatment modality for this group of patients.

**Materials and Methods:** We studied the effectiveness of a novel brain and VR treatment post-traumatic stress disorder (PTSD) in subjects who had suffered combat-related traumatic brain injuries in terms of PTSD symptom reduction. The trial was registered as ClinicalTrials.gov Identifier: NCT02003352. (http://clinicaltrials.gov/ct2/show/NCT02003352?term=carrick&rank=6). We analyzed the difference in the Clinician Administered DSM-IV PTSD Scale (CAPS) scores pre- and post-treatment using our subjects as their own matched controls. The study population consisted of 98 combat veterans maintaining an alpha of <0.05 and power of 80%.

**Results:** Prior to treatment, 75 subjects representing 76.53 % of the sample were classified in the 2 most severe categories of PTSD. Forty-one subjects, representing 41.80 % of the total sample, were classified in the extreme category of PTSD and 34 subjects, representing 34.70 % of the total sample, were classified in the severe category of PTSD. After treatment, we observed a large reduction in CAPS severity scores with both statistical and substantive significance.

**Discussion:** Treatment of PTSD as a physical injury rather than a psychiatric disorder is associated with strong statistical and substantive significant outcomes associated with a decrease of PTSD classification. The stigma associated with neuropsychiatric disorders may be lessened when PTSD is treated with brain and VR with a potential decrease in suffering of patients, family, and society.

## Introduction

Blast-related head injuries are among the most prevalent injuries suffered by military personnel deployed in combat (1) and mild traumatic brain injury (mTBI) or concussion on the battlefield in Iraq/Afghanistan has resulted in its designation as a “signature injury” (2). Blast-related mTBI appears to increase the risk of post-traumatic stress disorder (PTSD) with a substantial overlap between mTBI and PTSD issues (3, 4). Military deployments to Afghanistan and Iraq have been associated with an elevated prevalence of both PTSD and mTBI among combat veterans (5–7) The diagnosis and management of PTSD when a comorbid mTBI may also exist presents a challenge to interdisciplinary care teams at the Department of Veterans Affairs (VA) and civilian medical facilities, particularly when the patient reports a history of blast exposure (8). From a treatment perspective, trauma-focused cognitive behavioral therapy (TF-CBT) is the therapy that is associated with superior improvement in PTSD symptoms and psychosocial outcome in mTBI survivors (9) with a slight advantage compared to all other treatments (10). Vestibular complaints are the most frequent sequelae of mTBI, and vestibular rehabilitation (VR) has been established as the most important treatment modality for this group of patients (11). However, we could find no published case study reports or randomized controlled trials specific to VR in veterans who have PTSD after mTBI. Traumatic brain injury (TBI) caused by blast injury explosives used in modern warfare has become a common injury for troops, yet there is a paucity of neuropathology studies investigating the effects of high explosives on the human brain. There are, however, many studies that have demonstrated that athletes with repeated head trauma can develop a chronic traumatic encephalopathy (CTE), a neurodegenerative disease with similar clinical features to wartime blast injury (12). Our team has extensive experience in the treatment of mTBI and CTE in combat veterans, professional athletes, and Olympians using a novel VR. The PTSD Guideline Development Group and the National Collaborating Center for Mental Health review team has developed a guideline (NICE) based on best available evidence to advise on the treatment and management of post-traumatic stress disorder (PTSD) (13). NICE recommended three treatments [selective serotonin re-uptake inhibitors (SSRI), eye movement desensitization and reprocessing (EMDR) and TF-CBT] and a meta-analysis revealed that TF-CBT is most effective and associated with the best outcome (10, 14, 15). The reduction in PTSD symptoms is positively associated with a reduction in post-concussive symptoms suggesting that PTSD and mTBI symptoms are interdependent and mutually influence one another (16). Balance impairment, or postural instability, is a common source of residual physical disability after severe TBI (17) that is best treated with VR. Explosive blast TBI is one of the more serious wounds suffered by United States service members injured in the current conflicts in Iraq and Afghanistan (18) and there is limited research that demonstrates effective treatment outcomes when compared to the sports medicine literature of similar injuries. This investigation has the potential to change the treatment of PTSD after mTBI resulting in changed and saved lives of our service personnel and their families.

## Materials and Methods

### Research aims and hypothesis

#### Research question

The specific aim of this study was to study the effectiveness in terms of PTSD symptom reduction of a novel brain and VR treatment modality in patients with PTSD who have suffered combat-related traumatic brain injuries.

#### Hypothesis

Given the similarities in clinical features between CTE and blast-related mTBI, we hypothesized that VR treatment modalities will be effective in PTSD symptom reduction.

### Preliminary data

We searched a variety of databases for randomized controlled trials of mTBI and PTSD and VR up until September 2013 without success. Our search included Cochrane Injuries Group’s specialized register, Cochrane Depression, Anxiety and Neurosis Group’s specialized register, Cochrane Central Register of Controlled Trials, MEDLINE, PsycINFO, EMBASE, CINAHL, AMED, ERIC, and PsycBITE. We assembled an established clinical team of specialists, internationally known in the diagnosis and treatment of mTBI and PTSD to participate in this study. The 2008 Institute of Medicine review of interventions research for post-traumatic stress disorder (PTSD) concluded that new, well-designed studies are needed to evaluate the efficacy of treatments for PTSD (19). We had the funding and patient population to conduct and complete the study that will act as an initial pilot project. TBI may reflect an overlap between brain regions vulnerable to TBI, and the neural circuitry of these disorders (20).

### Research design and methods

The study was approved by our Institutional IRB and conducted in accordance with the principles of the Declaration of Helsinki. The trial was registered with a service of the U.S. National Institutes of Health as ClinicalTrials.gov Identifier: NCT02003352. There was equipoise.

### Study design

This before–after intervention trial was designed to identify the effectiveness of a novel brain and VR treatment modality in patients with PTSD who have suffered combat related traumatic brain injuries. Subjects served as their own matched controls. We accomplished the specific aim of our study by analyzing the difference in the Clinician Administered DSM-IV PTSD Scale (CAPS) scores (21) pre- and post-treatment in our subjects as outcomes to compare the effectiveness of the interventions. The CAPS is considered to be the gold standard for diagnosing PTSD and assessing symptom severity (21, 22). We expected differences in the outcomes after treatment to demonstrate a positive change that would be immediate and at follow-up over time, i.e., 3 months after cessation of a 2-week treatment period. All CAPS testing was conducted by one qualified licensed psychologist who was blinded to all components of the study. The study design included one pre-treatment assessment and two post-treatment assessments (at 1 week and 3 months). The study was performed at the department of Neurology of our Institutional Brain Center in Dallas, TX, USA. Patient recruitment began after IRB approval and study registration in October, 2013.

### Sample size

Our sample size calculations were based on expected differences in CAPS scores at follow-up. There is, however, a paucity of data in the literature specific to power and sample calculations over the long term. There are two quality randomized controlled studies that compared DSMIV CAPS score changes as outcome measures. One study looked at paroxetine therapy and the other TF-CBT. These studies demonstrated a total CAPS score of 34.8 points (SD = 25.7) in the paroxetine group (23) and 23.7 (SD = 26.1) in the cognitive behavioral group (24). There are no studies that measure changes in CAPS scores after VR. We estimated that the VR group in our study would have at least a comparable outcome to the paroxetine group. The power calculations demonstrated that a total of 89 participants were needed to demonstrate a difference of 11.1 points (alpha = 5%, power = 80%). A difference of 0.50 SD represents a decrease in CAPS scores of approximately 10 points. We felt that an effect size (ES) of *d* = 0.50 would represent a clinically significant effect. The estimated sample size for the Cox PH regression Wald test, log-hazard metric in a 2-group comparison demonstrated that 66 subjects would be needed. Further, the estimated sample sizes for two-sample comparison of survivor functions using the Log-rank test, Freedman method with an alpha of 0.05 (two sided) and a hazard ratio of 0.50 and 80% power demonstrated that we needed a total of 72 subjects. All of these sample size calculations were similar and we therefore decided that 82 subjects would be needed in total. To allow for a 20% attrition rate at follow up, we added an additional 16 subjects to the study for a total of 98 subjects. This study design maintained a Type I error at an acceptable level of 0.05 in order to minimize the risk of false positive findings.

### Participants

The study population consisted of 98 combat veterans who had suffered a TBI with PTSD, referred to our study by Veteran’s groups. All subjects were male with a mean age of 39 years with a minimum age of 20 years and a maximum age of 60 years. They all met the inclusion requirements and did not have any of the exclusion requirements.

### Inclusion criteria

Military combat veterans who had suffered a TBI and post-traumatic stress disorder (PTSD) that were exposed to war-zone events in operation enduring freedom (OEF) and/or operation Iraqi freedom (OIF). Subjects fulfilled all criteria for a diagnosis of chronic PTSD based on the DSM-IV (25) with qualifying scores on the CAPS (21). All subjects must have had previous treatments for PTSD that were not successful. Subjects were 18 years of age or older and were able to give written informed consent.

### Exclusion criteria

Presence of any of the following DSM-IV diagnoses: psychotic disorder, mania or bipolar disorder; current major depression with psychotic features; current drug or alcohol or substance/drug dependence; patients who are considered a suicidal risk.

### Assessments and outcome measures

We used changes in the DSM-IV CAPS scores before and after treatment to distinguish between the estimated frequency and intensity of the various symptoms. Frequency and intensity scores were combined to give a total CAPS score (range: 0–136) as standard in the DSM-IV CAPS evaluation procedures (21, 22). CAPS testing was scheduled pre-intervention, 1 week post-intervention, and at 3 months post-intervention.

### Intervention: Brain and vestibular rehabilitation

Each subject was treated with strategies central to gaze stabilization with head movements and activation of the vestibular–ocular response, off axis whole body rotation, visual pursuit, and visual saccadic eye movements to novel targets [well described in Ref. (26–30)]. However, because our clinical experience has shown that customized treatment based on reported symptoms and finding of physical and neurological examination by trained clinicians is more effective than standard VR treatment modalities in athletes suffering repeated concussions, each subject received a custom treatment plan. This allowed the doctor to tailor the treatment and maximize its effect. For example, one subject might have a deficit of gaze holding in right gaze and another in left gaze. Both would have gaze holding strategies prescribed specific to their clinical needs. To avoid inter-rater variability, the same clinician decided the treatment plan for all subjects. Each subject received daily sessions of three VR treatment modalities for 2 weeks (5 week days per week with 2 weekend days off). Subjects were instructed to rest between treatments. No medication changes were prescribed during the treatment period. The treatment was administered by clinicians certified in VR. These clinicians did not know the results of the CAPS pre-treatment evaluation.

### Procedure

All subjects met the inclusion criterion at the referring agency and then underwent a comprehensive medical history and neurological examination as well as a CAPS test to confirm PTSD after mTBI. They were carefully examined to ensure that they did not meet any of the exclusion criteria. The subjects that were acceptable to the study were given a detailed explanation of the study and an offer to participate in the study after giving informed consent. Participants underwent another CAPS test 1 week after their treatment had been finished and were scheduled again at 3 months.

### Statistical analyses

A statistical analysis was conducted using Stata/SE 13.1 (StataCorp, College Station, TX, USA) according to an intention-to-treat (ITT) approach. Since it was expected that some participants might not complete the study for a variety of reasons, the analysis procedure included provisions for identifying these individuals making a careful note of the reasons for no completion if possible. However, compliance with treatment appointments was necessary for the subject to be included in the analysis. An individual who missed more than 25% of their treatment appointments would be categorized as being non-compliant. Dropouts were to be identified separately from those individuals who were deemed non-compliant. The short-term efficacy of the treatment modality was evaluated by considering the difference pre and post (at 1 week after treatment) of the CAPS Total Severity Scores for each subject (matched pairs) and by calculating the probability of error (*p* value) by a two-tailed *t*-Test for repeated measures maintaining an alpha of <0.05. The ES was calculated in three ways to ascertain if the difference between the matched pairs was both statistically and clinically significant: as proposed by Cohen, the ratio of the mean difference between the two groups (pre and 1 week post) divided by the pooled variance of the groups was calculated (31) Another ES as proposed by Hedges was calculated using a formula similar to Cohen’s but calculating the SD using *N* − 1 instead of *N* (where *N* is the number of samples considered) (32). In both cases, an ES value of 0.2 represents a small statistical and clinical difference between two groups; an ES value of 0.5 represents a moderate difference; and an ES value of 0.8 represents a large difference (31). Finally, the ES was obtained by calculating the point biserial correlation: a percent improvement between CAPS Total Severity Score was calculated [(post-test group mean minus pre-test group mean) divided by (pre-test group mean)] × 100), the changes between sequential CAPS scores were measured and how strong the relationship was between them was calculated. In this last case, a value of 0.01–0.09 is a small effect, a value of 0.10–0.25 is a medium effect and a value of over 0.25 represents a large ES. Similar calculations were done to assess the long-term efficacy of the treatment modality by considering the difference pre and post (at 3 months after treatment) of the CAPS Total Severity Scores for each subject (matched pairs).

## Results

**Figure 1:**
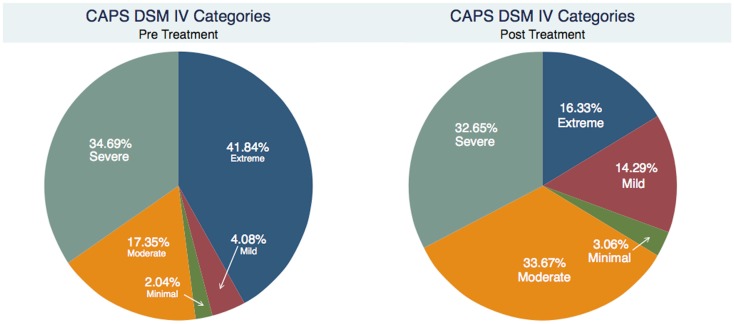
**Percentage of CAPS total severity scores pre and 1 week post-treatment for each category (Minimal, Mild, Moderate, Severe, and Extreme)**.

**Figure 2:**
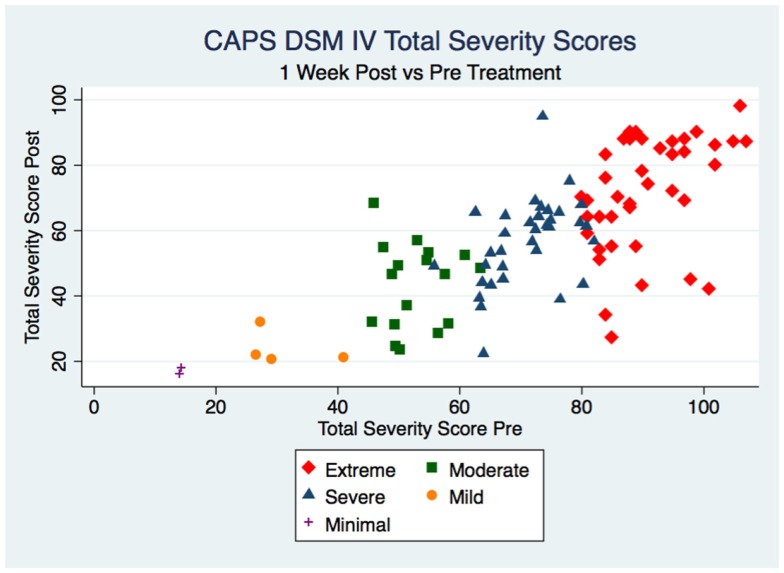
**Scatter plot of 1 week post- vs. pre-treatment scores color coded for the different categories**.

For the 98 subjects participating in the study, Table 1 shows the two-way table with measures of association for the CAPS Total Severity Scores pre- and 1 week post-treatment divided into each category (Minimal, Mild, Moderate, Severe, and Extreme) and their relative percentage. The rows represent the pre-treatment classification, the columns represent the 1 week post-treatment classification. Figure 1 gives a graphical representation of the same percentages, whereas Figure 2 shows the scatter plot of the 1 week post-treatment CAPS Total Severity Scores versus the pre-treatment CAPS Total Severity Scores for each subject, color coded for the different categories pre-treatment. The change in CAPS Total Severity Scores had a χ^2^ (16, *N* = 98) = 132.7399, *p* < 0.001, ϕ = 0.5819.

**Table 1:** **Two-way table with measures of association for the CAPS Total Severity Scores pre and 1 week post-treatment divided into each category (Minimal, Mild, Moderate, Severe, and Extreme), and their relative percentage**.

Severity category pre	Severity category post					
	Minimal	Mild	Moderate	Severe	Extreme	Total
**Minimal 0–19**	2	0	0	0	0	2
	100.00	0.00	0.00	0.00	0.00	100.00
	66.67	0.00	0.00	0.00	0.00	2.04
**Mild 20–39**	0	4	0	0	0	4
	0.00	100.00	0.00	0.00	0.00	100.00
	0.00	28.57	0.00	0.00	0.00	4.08
**Moderate 40–59**	1	7	8	1	0	17
	5.88	41.18	47.06	5.88	0.00	100.00
	33.33	50.00	24.24	3.12	0.00	17.35
**Severe 60–79**	0	1	17	15	1	34
	0.00	2.94	50.00	44.12	2.94	100.00
	0.00	7.14	51.52	46.88	6.25	34.69
**Extreme 80–136**	0	2	8	16	15	41
	0.00	4.88	19.51	39.02	36.59	100
	0.00	14.29	24.24	50	93.75	41.84
**Total**	3	14	33	32	16	98
	3.06	14.29	33.67	32.65	16.33	100.00
	100.00	100.00	100.00	100.00	100.00	100.00

*χ^2^ (16, *N* = 98) = 132.7399, *p* < 0.001, ϕ = 0.5819*.

*The rows represent the pre-treatment classification, the columns the 1 week post-treatment classification*.

Table 2 shows, for the five pre-treatment categories of the CAPS Total Severity Scores and for the overall population, the mean and its 95% confidence interval (95% CI), the standard error, and the SD of the pre, 1 week post, and difference distributions, as well as the two-tailed repeated measures pre–post *t*-Test, its significance *p*, and the three calculated ES (Point-biserial *r*, Cohen’s *d*, and Hedge’s *g*) based on mean comparison and their 95% CI.

**Table 2:** **For the different pre-treatment categories of the CAPS Total Severity Scores and for the overall population: mean and its 95% confidence interval (CI), standard error, and standard deviation of the pre, 1 week post, and difference distributions, as well as the two-tailed repeated measures pre–post *t*-Test, its significance *p*, and the three calculated effect size (Point-biserial *r*, Cohen’s *d*, and Hedge’s *g*) based on mean comparison**.

		Mean (95% CI)	SE	SD	*t*	*P*	Point-biserial *r* (95% CI)	Cohen’s *d* (95% CI)	Hedges’s *g* (95% CI)
Minimal (2 sbj)	Pre	15.00 (−35.82 65.82)	4.00	5.66	0.67	0.626	−0.32 (−0.86 0.74)	−0.49 (−2.44 1.58)	−0.27 (−1.38 0.89)
	1 week Post	17.00 (4.29 29.71)	1.00	1.41	
	Diff	2.00 (−40.12 36.12)	3.00	4.42	
Mild (4 sbj)	Pre	31.00 (21.81 40.19)	2.89	5.77	1.68	0.192	0.51 (−0.28 0.82)	1.04 (−0.50 2.50)	0.90 (−0.43 2.17)
	1 week Post	25.50 (17.87 33.13)	2.40	4.80	
	Diff	5.50 (−4.93 15.93)	3.28	6.56	
Moderate (17 sbj)	Pre	52.41 (50.06 54.77)	1.11	4.58	2.38	**<0.05**	0.40 (0.07 0.62)	0.84 (0.13 1.53)	0.82 (0.13 1.50)
	1 week Post	44.00 (37.09 50.91)	3.26	13.44	
	Diff	8.41 (0.93 15.92)	3.54	14.60	
Severe (34 sbj)	Pre	70.65 (68.65 72.64)	0.98	5.71	7.05	**<0.001**	0.56 (0.38 0.69)	1.33 (0.80 1.85)	1.32 (0.79 1.83)
	1 week Post	56.88 (52.19 61.58)	2.31	13.45	
	Diff	13.76 (9.79 17.74)	1.95	11.38	
Extreme (41 sbj)	Pre	90.73 (88.36 93.10)	1.17	7.51	7.87	**<0.001**	0.60 (0.44 0.70)	1.47 (0.97 1.95)	1.45 (0.97 1.93)
	1 week Post	71.20 (65.74 76.65)	2.70	17.27	
	Diff	19.54 (14.56 24.51)	2.46	15.77	
Total (98 sbj)	Pre	73.13 (69.17 77.09)	2.00	19.76	9.98	**<0.001**	0.35 (0.22 0.46)	0.73 (0.44 1.02)	0.73 (0.44 1.02)
	1 week Post	58.54 (54.52 62.56)	2.02	20.03	
	Diff	14.45 (11.53 17.37)	1.47	14.49	

*In bold, the significant values*.

## Discussion

**Figure 3:**
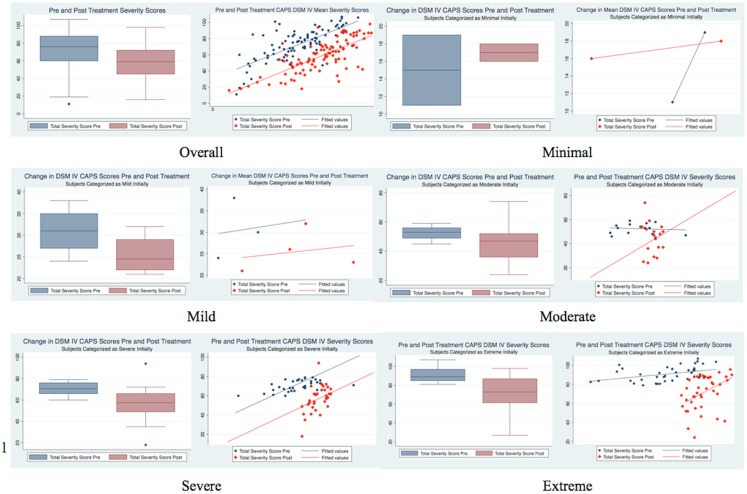
**Box plots and scatter plots of pre and 1 week post-treatment for the different pre-treatment categories of the CAPS total severity scores and for the overall population, obtained from the results of Table 2**. When considering the 3 months follow up, 84 subjects dropped out of the study. For the 14 that underwent the CAPS testing, the mean CAPS Total Severity Score was 53.14.

As indicated in Table 1 and Figure 1, prior to treatment, 75 subjects (representing 76.5% of the sample) were classified in the two most severe categories of PTSD: of these, 41 subjects (41.8%) were classified in the Extreme category, and 34 subjects (34.7%) were classified in the Severe category; 17 subjects (17.4%) were classified in the Moderate category; 4 subjects (4.1%) were classified in the Mild category, and only 2 subjects (2.0%) were classified in the Minimal category. One week after treatment, only 48 subjects (49.0%) remained in the two most severe categories of PTSD: this represents a 36% improvement of the symptoms. The improvement effect is even more remarkable when considering the Extreme category: 41 subjects pre-treatment vs. 15 1 week post-treatment, a success rate in decreasing the symptoms classification of 63.4%. Furthermore, the improvement allowed subjects to be classified not only in the Severe category (the immediate lower category) but also in the Moderate and Mild categories: 16 subjects (39.0%) were reclassified as Severe sufferers post-treatment, 8 subjects (19.5%) as Moderate, and 2 subjects (4.9%) as Mild. Similar trend was found for subjects originally classified in the Severe category [of those 34 subjects, 15 (44.1%) remained in the Severe category, 17 (50%) became Moderate, and 1 (2.9%) became Mild] and in the Moderate category [of those 17 subjects, 8 (47.1%) remained in the Moderate category, 7 (41.2%) became Mild and 1 (5.9%) became Minimal]. In both cases, only 1 subject (2.9% for the Severe category and 5.9% for the Moderate category respectively) had worse symptoms classification 1 week post-treatment. The subjects classified in the Mild and Minimal categories maintained the same classification 1 week post-treatment. Furthermore, the overall results showed in Table 1 revealed strong statistical (*p* = 0.001 < 0.05) and substantive (ϕ = 0.58 > 0.5) significance (a ϕ >0.5 is considered a strong relationship). Considering Figure 2, the same information can be extrapolated: for example, the two subjects in the minimal category pre-treatment (with pre-treatment score <20 – *x* axis) stayed in the same category 1 week post-treatment (their *y* value is still <20); similarly, for the 34 subjects in the Severe category (pre-treatment score between 60 and 80), 1 had an increase in score (higher than 80), 15 stayed in the same range, 17 moved to the immediately lower range (40–60), and 1 “jumped” two categories lower (20–40). When considering how much change each subject had (Figure 3), as well as on average by categories, it is evident that the “worse” the original classification, the greater the change (a negative change indicates improvement, a positive change indicates a worsening of the condition). Furthermore, with the exclusion of two subjects that had a worsening of the classification of their symptoms after treatment (one in the Severe and one in the Moderate category as Reported in Table 1), most of the subjects, even if they did not change category, showed an improvement: only four subjects, i.e., 4.1% (one in the Mild, two in the Moderate, and one in the Severe), had a clear worsening of their CAPS scores. Furthermore, these changes are statistically significant for the Moderate, Severe and Extreme categories (*p* < 0.05 as indicated in Table 2). They are also substantively significant as indicated by the ES reported in Table 2 as well. For the Extreme category not only do all three ES indicate a large effect (*r* > 0.25, and *d* and *g* > 0.8), with their 95% CI also demonstrating this. For the Severe category the same applies except for the lower limit of the Hedge’s *g* CI. For the Moderately category, the ES indicate a large effect, but not when considering their 95% CI. The Mild and Minimal categories have too few subjects that their results are not statistically significant (*p* > 0.05), although for the Mild category, the ES is still large (but not when considering their 95% CI). When considering all the subjects together, the changes are statistically significant (*p* < 0.001), but only the point-biserial *r* indicates a substantive significance (*r* = 0.35 > 0.25) almost with 95% confidence (lower limit = 0.22, thus medium effect), whereas the Cohen’s *d* and Hedge’s *g* indicate a moderate ES (0.73 < 0.8).

One more consideration must be made regarding the subjects in our study: they had a mean pre-treatment CAPS Total Severity Score equal to 210% of that reported in the paroxetine therapy (23) study (73.1 vs. 34.8) and to 308.4% of that reported in the cognitive behavioral study (24) (73.1 vs. 23.7). These higher scores represented greater levels of PTSD and yet the outcomes of our treatment strategies have been successful and associated with a greater statistical and substantive significance than we hypothesized. Unfortunately, only 14 subjects returned for the 3-month follow up CAPS evaluation: after treatment, most subjects were able to return to a higher quality of normal life with increased activity to become productive members of society that did not feel the need to be assessed again. Traveling time and costs could have also played a role in this decision not to return for the follow-up evaluation as the subjects were sent to us from around the United States. Although the significance of the 3-month post-treatment results is not as strong as the results obtained at the 1-week post-treatment, there was still a mean improvement (53.14 vs. 58.54 after 1 week) pointing toward a possible continuing improvement of the PTSD symptoms. When considering the overall results obtained in this study, the greater improvements found with subjects in the Extreme and Severe categories are in line with what we usually find in our clinical experience with athletes suffering from CTE and mTBI: the worse the symptoms, the greater the response to the customized novel brain and VR treatment modality used in this study. This appears to follow the Pareto Principle or 80–20 curve, for which there is a lot of improvement for the same amount of effort if the margin for improvement is large. In this particular situation, subjects in the Extreme and Severe categories had so many issues and symptoms that treatment had larger effects. This also confirms what we see in our clinical experience.

## Conclusion

This investigation has analyzed the use of a novel brain and VR treatment modality in PTSD patients who have suffered combat-related traumatic brain injuries immediately and over time after treatment. In general, we obtained both strong statistical and substantive significant outcomes. The treatment of this disorder as a physical injury with brain and vestibular non-invasive and non-pharmaceutical applications has never been reported. Further, a successful 2-week treatment period may be associated with significant savings of cost, time, and disability when compared to longer therapy programs. There may be a stigma associated with having a neuropsychiatric diagnosis of PTSD that might be lessened if a physicality of etiology similar to that commonly accepted in mTBI is embraced. Our investigation has the promise of development of superior outcomes of treatments in this area that will benefit a global society.

### Strengths and weaknesses of the study

Clinical practice guidelines are dependent upon quality investigations that ultimately drive physician applications. We are embracing an opportunity to be involved in change and best practice development associated with the positive outcome of our study. The observed clinical results of our treatments might change the direction of therapy sponsored by government and private agencies. We may have a limitation of our study specific to the gathering of long-term post-treatment CAPS scores because of the nation wide distribution of our sample population as well as life style changes associated with decreased PTSD suffering. We have had difficulty having subjects return to our facility for retesting and we are designing a better method of obtaining follow-up testing for future investigations. We utilized only one licensed psychologist trained in administration of the CAPS in order to decrease any inter-examiner differences. We are considering having multiple psychologists administer the CAPS testing at different locations throughout the country and controlling for inter-examiner differences in the future, so to facilitate the return of subjects for long-term follow-up evaluation. We are also looking into the inclusion of other instruments to measure subject satisfaction and depression as another possible measure of outcome measure. This study did not include any female subjects. We are aware of many female military veterans who have suffered blast injuries and resultant PTSD but no females were assigned to us by the referring agencies. Future research is needed to verify if similar outcomes can be obtained with women.

## Conflict of Interest Statement

The authors declare that the research was conducted in the absence of any commercial or financial relationships that could be construed as a potential conflict of interest.
